# Structural Aspects of the Antiparallel and Parallel Duplexes Formed by DNA, 2’-O-Methyl RNA and RNA Oligonucleotides

**DOI:** 10.1371/journal.pone.0143354

**Published:** 2015-11-18

**Authors:** Marta Szabat, Tomasz Pedzinski, Tomasz Czapik, Elzbieta Kierzek, Ryszard Kierzek

**Affiliations:** 1 Institute of Bioorganic Chemistry, Polish Academy of Sciences, Noskowskiego 12/14, 61-704 Poznan, Poland; 2 Department of Chemistry, Adam Mickiewicz University, Umultowska 89B, 61-614 Poznan, Poland; The University of Queensland, AUSTRALIA

## Abstract

This study investigated the influence of the nature of oligonucleotides on the abilities to form antiparallel and parallel duplexes. Base pairing of homopurine DNA, 2’-O-MeRNA and RNA oligonucleotides with respective homopyrimidine DNA, 2’-O-MeRNA and RNA as well as chimeric oligonucleotides containing LNA resulted in the formation of 18 various duplexes. UV melting, circular dichroism and fluorescence studies revealed the influence of nucleotide composition on duplex structure and thermal stability depending on the buffer pH value. Most duplexes simultaneously adopted both orientations. However, at pH 5.0, parallel duplexes were more favorable. Moreover, the presence of LNA nucleotides within a homopyrimidine strand favored the formation of parallel duplexes.

## Introduction

DNA exists as a right-handed, double-stranded structure, which is classified as a B helix. RNA also appears as a right-handed structure (A helix). However, in native RNA, double-stranded helical regions are short (on average 5–7 base pairs long). Moreover, RNA contains many structural motifs disrupting its helical structure, such as various types of single and multinucleotide mismatches, internal loops, hairpins, bulge loops, terminal unpaired regions and multibranch [1]. Some specific sequences under certain conditions can form a left-handed helical structure [2]. The transition from one structure to another occurs depending on the base sequence, humidity, type and concentration of cations and anions, temperature and pH value. Although double helical structures are the most common structures, nucleic acids can form triplexes or quadruplexes in parallel and antiparallel strand orientations [3–5].

Interacting strands in helical DNA and RNA exist in antiparallel orientation (5’-3’/3’-5’), and they are stabilized by Watson-Crick hydrogen bonds as well as intra- and/or inter-strand stacking interactions. Conversely, DNA and RNA can adopt a parallel-stranded (5’-3’/5’-3’) structure, which is an unusual duplex form [6].

Pattabiraman suggested that the formation of parallel DNA duplexes occurs by reverse Watson-Crick base pairs, also called Donohue base pairs, and they confirmed the formation of this structure by Raman spectroscopy, NMR spectroscopy and chemical methylation [7]. At neutral pH, the parallel duplex structure is stabilized by Donohue type A-T and G-C base pairs. However, some of the hydrogen bonds require protonation of heterocyclic bases, thus causing pH-dependent parallel duplex formation [8, 9].

Interestingly, parallel-stranded purine-pyrimidine stretches have been found in different genomes, and these stretches may have an evolutionary role [10]. For example, the Chernov group described a region of parallel DNA fragments in the *Drosophila melanogaster* genome [11]. Such parallel duplexes have also been detected in mRNA of the *lon* gene in *E*. *coli* [12]. Moreover, sequences with a propensity to form parallel strands can be templates for the formation of triplexes, quadruplexes and H-type structures [13–16]. Parallel structures of DNA and RNA can be promising tools in molecular genetics, and they can also be used as antisense oligonucleotides or aptamers in the regulation of gene expression [17, 18].

There have been several theoretical and experimental reports on the formation of parallel DNA duplexes, but there has been no data reported on parallel structures of natural and modified RNA/RNA and DNA/RNA duplexes. Parallel duplexes have been characterized by thermodynamic and spectroscopic analysis as well as by a variety of chemical and biochemical methods [19–22]. As numerous studies have shown for unmodified oligonucleotides, the thermal stability of parallel duplexes is significantly lower in comparison to that of isosequentional antiparallel duplexes [23, 24]. Moreover, the formation of stable parallel duplexes requires specific sequences, acidic conditions and low temperature, which are different from those existing in cells. A number of chemical modifications have been developed to increase the thermodynamic stability of parallel duplexes under physiological conditions. One group of nucleotides that stabilize parallel-stranded duplexes includes base modifications, such as 8-amino-2’-deoxyadenosine [25], 8-amino-2’-deoxyguanosine [26] and 2’-deoxy-5-methyloisocytidine [27] as well as 8-amino-2’-deoxyguanosine with a modified ribose moiety (*i*.*e*., 2’-O-MeRNA or locked nucleic acid) [28, 29].

**Fig 1:**
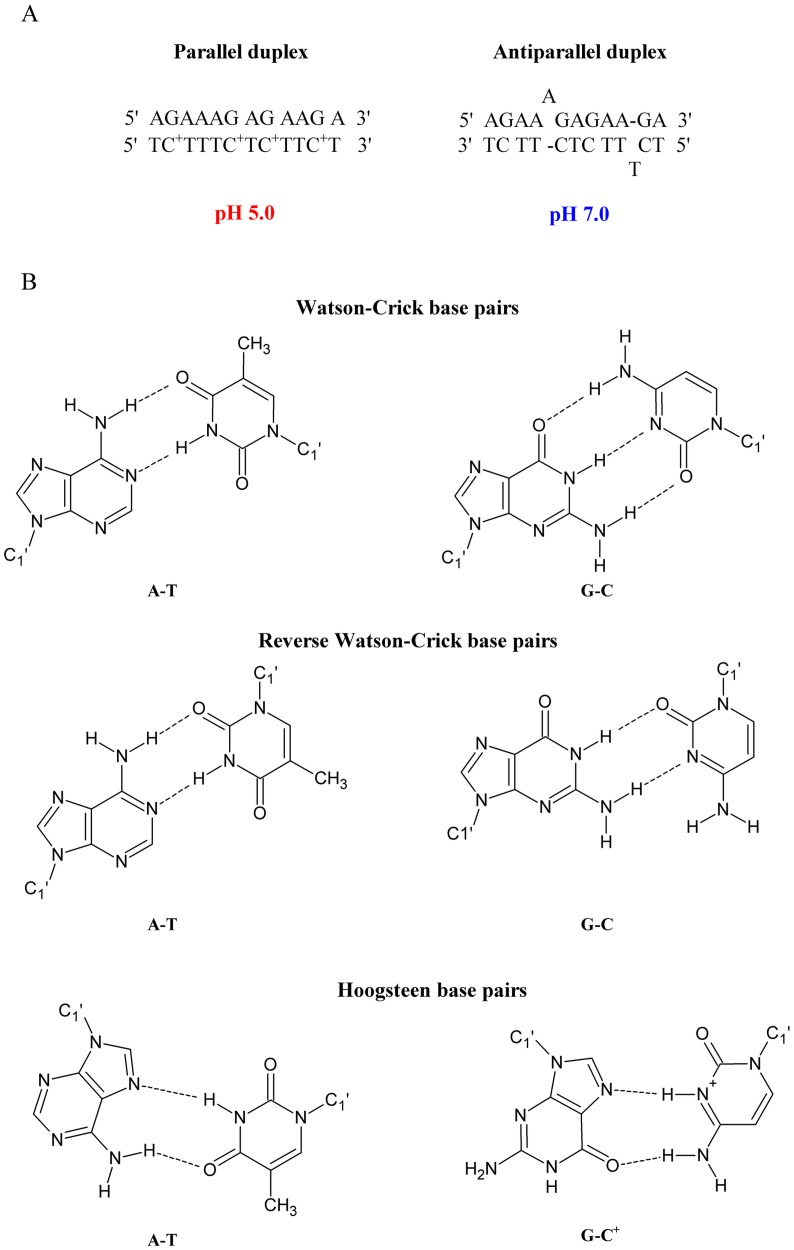
Parallel and antiparallel-stranded DNA duplexes depending on pH value (A) and chemical structure of the Watson-Crick and reverse Watson-Crick as well as Hoogsteen base pairs (B).

This study presents the thermodynamic and structural details of a series of unmodified and modified DNA, 2’-O-MeRNA and RNA homo- and heteroduplexes. A homoduplex is a duplex that is formed by the same type of strands (e.g., DNA or RNA), whereas a heteroduplex is a duplex formed by various types of strands (e.g., one strand is DNA and the second strand is RNA). The model duplexes were designed accordingly to previously published paper [29]. The parallel duplex was arranged to form fully matched base pairs and for the formation of this duplex it is necessary protonation of cytosine bases at acidic pH. Parallel DNA duplex is stabilized by Hoogsteen hydrogen bonds, whereas at neutral pH, the antiparallel duplex is formed with Watson-Crick base pairings and two bulges (A and T) (Fig 1). To confirm parallel and antiparallel orientations of model duplexes, many biophysical studies, including steady-state fluorescence, time-resolved fluorescence, UV melting and circular dichroism (CD) spectroscopy, were performed. The data, particularly fluorescence resonance energy transfer (FRET) measurements, suggested that 2’-O-MeRNA and RNA duplexes form parallel duplexes at pH 5.0 and 7.0. Furthermore, incorporation of LNA moieties favored parallel duplex formation at pH 7.0.

## Materials and Methods

### Synthesis and purification of oligonucleotides

All oligonucleotides were synthesized on a MerMade12 (BioAutomation) synthesizer using standard phosphoramidite chemistry [30]. Commercially available RNA, DNA, 2’-O-MeRNA and LNA phosphoramidites (Glen Research, GenePharma, Exiqon) were used for synthesis. TAMRA-labeled oligonucleotides were synthesized from an oligonucleotide with a 5’-amino-modifier-C6 due to conjugation with the TAMRA N-hydroxysuccinimide active ester. FAM-labeled oligonucleotides were synthesized using fluorescein-labeled phosphoramidite. The details of deprotection and purification of oligonucleotides have been described previously [31, 32]. Purified oligonucleotides were characterized using MALDI-TOF mass spectrometry.

### UV melting experiments

Oligonucleotides were melted in a buffer containing 40 mM boric acid, 40 mM phosphoric acid, 40 mM acetic acid and 100 mM sodium chloride adjusted to pH 5.0 or 7.0 with 0.2 M sodium hydroxide (Robinson Britton buffer) [29]. Oligonucleotide single-strand concentrations were calculated from the absorbance measured at a temperature above 80°C, and single-strand extinction coefficients were approximated by a nearest-neighbor model. LNA and 2’-O-MeRNA strands with identical sequences were assumed to have identical extinction coefficients. Absorbance versus temperature melting curves were recorded at 260 nm with a heating rate of 0.5°C/min from 2 to 90°C on a Beckman DU 640 with a thermoprogrammer. Before the measurement, the samples were denatured for 2 min at 90°C and then slowly cooled to room temperature overnight. Melting curves were analyzed, and the thermodynamic parameters were calculated from a two-state model using MeltWin 3.5 software [33]. For most duplexes, the ΔH° derived from T_M_^-1^ vs. ln(C_T_/4) plots was within 15% of the value derived from averaging the fits to individual melting curves as expected if the two-state model is reasonable.

### Circular dichroism

CD spectra were recorded using a JASCO J-815 spectropolarimeter at 4°C in 0.2 cm path length quartz cuvettes. The oligonucleotides were dissolved in RB buffer (pH 5.0 or 7.0) to achieve 4.4 μM sample concentration. Before the measurement, the samples were denatured for 2 min at 90°C, slowly cooled at room temperature and incubated at 4°C overnight. The measurements were taken in triplicate in the 200–350 nm wavelength range with a 1 nm data interval. The CD curves were established as an average of three CD scans. The spectrum of the buffer was subtracted, and the results were converted into molar ellipticity per nucleotide (Δε).

### Fluorescence spectroscopy

To determine the relative orientation of the strands in the duplex, the energy transfer between FAM and TAMRA was measured. Two types of fluorescence experiments, *i*.*e*., steady-state and time-resolved fluorescence measurements, were performed.

### Steady-state fluorescence measurements

Each duplex was dissolved in RB buffer to achieve a 4.4 μM sample concentration. All samples were denatured at 90°C for 2 min and then slowly cooled to room temperature overnight. All measurements were carried out in the 450–650 nm wavelength range with a 1.5 nm data interval. FRET assays were performed at 10°C. Steady-state fluorescence spectra were measured and imaged using a FluoTime 300 EasyTau spectrometer (PicoQuant, Germany).

### Time-resolved fluorescence measurements

All samples were prepared as described above for the steady-state fluorescence measurements and were performed with excitation at 440 nm. FRET assays were performed at 10°C. Fluorescence decays were measured by the time-correlated single photon counting technique (TCSPC) using a FluoTime 300 EasyTau spectrometer (PicoQuant). Fluorescence decay curves were analyzed using FluoFit Analysis software (PicoQuant). The values of lifetimes (*τ*_*D*_, *τ*_*DA*_) for DNA and RNA duplexes were calculated using the Lorentzian lifetime distribution model with reconvolution. The quality of the fit was judged on the basis of the chi-squared parameter (understood as χ^2^ ≤ 1.2).

## Results

### Thermodynamic features of duplexes

Thermodynamic parameters were calculated, using the program MeltWin 3.5, by two methods: (i) from fits of individual melting curves at nine different duplex concentrations, (ii) plots of reciprocal melting temperatures (*T*_m_^–1^ versus ln*C*_T_/4). The two-state transition was assumed to be valid for duplexes in which the agreement in ΔH° values of the two methods is within 15%. The error-weighted (standard deviation) average of the data obtained by the two methods was calculated and reported in S1 Table.

Thermodynamic data for model DNA and RNA duplexes are summarized in S1 Table. One strand was composed by purine and second one by pyrimidine nucleotides. Moreover, some pyrimidine oligonucleotides contained LNA-cytidine. Model duplexes were formed by the same (DNA/DNA, RNA/RNA and 2’-O-Me RNA/2’-O-Me RNA, homoduplexes) or various (DNA/RNA, DNA/2’-O-Me RNA, RNA/2’-O-Me RNA, heteroduplexes) type of strands. Analysis of melting curves at 260 nm in RB buffer pH 7.0 and thermodynamic parameters demonstrated that most duplexes melt according to two-state transition. It concerns RNA and 2’-O-Me RNA duplexes, however, for DNA heteroduplexes (S1 Table, D3-D6) melting transition was mostly non-two-state. The similar observation concerns melting of the duplexes in RB buffer at pH 5.0. DNA duplexes D1-D6 melted according to non two-state transition, whereas remaining RNA and 2’-O-Me RNA duplexes (except D9 and D16) melted according to two-state transition (S1 and S2 Figs).

Collected thermodynamic data demonstrate that type of strands, the presence of LNA modification within one strand and pH of RB buffer have influence on character of melting and thermodynamic stabilities of model D1-D18 duplexes [34]. For duplexes D1-D6 with 5’d(AGAAAGAGAAGA) pairing with DNA (D1), DNA-LNA (D2), RNA (D3), RNA-LNA (D4), 2’-O-Me RNA (D5) and 2’-O-Me RNA-LNA (D6) melting of most duplexes was non two-state, both at pH 5.0 and 7.0. The exceptions were duplexes D1, D2 and D5 at pH 7.0. Comparison of the thermodynamic stabilities of duplexes was studied by free energy calculated from the correlation between melting temperature and duplex concentration (1/T_M_ vs ln C_T_/4). Presence of three LNA-cytidine residues enhances stabilities of duplexes in range of 1.8–2.7 kcal/mol, both in buffer pH 5.0 and 7.0. The exception was duplex D2 at pH 7.0 where LNA residues diminished stability by 0.6 kcal/mol (S1 Table). Comparison of the thermodynamic stabilities of the duplexes formed by 5’d(AGAAAGAGAAGA) in buffer pH 5.0 and 7.0 indicates that they are more stable in buffer pH 5.0 by 1.6, 4.0, 3.5, 4.2, 4.3 and 4.5 kcal/mol for duplexes D1, D2, D3, D4, D5 and D6, respectively. For series RNA and 2’-O-Me RNA, 5’r(AGAAAGAGAAGA) or its 2’-O-methylated analog were paired with DNA, DNA-LNA, RNA, RNA-LNA, 2’-O-Me RNA, 2’-O-Me RNA-LNA and form duplexes D7-D12 and D13-D18, respectively (Table 1). Incorporation three LNA-C in those duplexes enhances stabilities by 2.1 and 3.4 kcal/mol, on average. In contrast to D1-D6, the duplexes D7-D18 are more thermodynamically stable by 0.3–1.9 kcal/mol in buffer pH 7.0 than pH 5.0. The exception is duplex D14 which is ca. 0.4 kcal/mol more stable at pH 5.0.

**Table 1:** Summary of biophysical properties of D1-D18 duplexes at various buffers pH values.^a^

Sequences of duplexes and control oligonucleotides	Name of duplexes and control probes	Thermodynamic stabilities			Fluorescence measurements^b^						Analysis of CD spectra	
		pH 5	pH 7		pH 5			pH 7			pH 5	pH 7
		-ΔG°_37_ (kcal/mol)	-ΔG°_37_ (kcal/mol)	ΔΔG°_37_ (pH 5)–ΔΔG°_37_ (pH 7)	Lifetime *τ*_*DA*_ (ns)	FRET efficiency *E* (%)	Distance *R* (Å)	Lifetime *τ*_*DA*_ (ns)	FRET efficiency *E* (%)	Distance *R* (Å)	h_1/2_	h_1/2_
AGAAAGAGAAGA	S1*	-	-	-	2.5 ^τD^	0	*R*_*0*_	3.8	0	*R*_*0*_	-	-
AGAAAGAGAAGA/TCTTTCTCTTCT	D1	6.25	4.67	-1.58	2.4	2	104	3.4	10	78	-0.50	3.25
AGAAAGAGAAGA/TC^L^TTTC^L^TCTTC^L^T	D2	8.07	4.11	-3.96	2.5	0	>108	3.2	16	70	1.05	1.55
AGAAAGAGAAGA/*UCUUUCUCUUCU*	D3	6.77	3.27	-3.50	2.5	0	>108	3.7	2	106	-0.40	2.00
AGAAAGAGAAGA/*UC*^*L*^*UUUC*^*L*^*UCUUC*^*L*^*U*	D4	9.42	5.26	-4.16	2.5	0	>108	3.7	2	106	-0.95	0.40
AGAAAGAGAAGA/U^M^C^M^U^M^U^M^U^M^C^M^U^M^C^M^U^M^U^M^C^M^U^M^	D5	7.70	3.42	-4.28	2.4	2	103	3.8	0	>108	-0.50	1.10
AGAAAGAGAAGA/U^M^CLU^M^U^M^U^M^C^L^U^M^C^M^U^M^U^M^C^L^U^M^	D6	10.35	5.81	-4.54	2.5	0	>108	3.8	0	>108	-3.30	0.00
*AGAAAGAGAAGA*	S2*	-	-	-	2.4 ^τD^	0	*R*_*0*_	3.7	0	*R*_*0*_	-	-
*AGAAAGAGAAGA*/TCTTTCTCTTCT	D7	6.44	7.12	0.68	1.8	26	64	1.7	55	52	-1.50	0.90
*AGAAAGAGAAGA*/TC^L^TTTC^L^TCTTC^L^T	D8	9.18	9.94	0.76	1.4	57	57	1.2	68	47	-1.25	0.45
*AGAAAGAGAAGA/UCUUUCUCUUCU*	D9	7.58	7.99	0.41	1.6	66	48	0.4	89	38	-2.05	-0.15
*AGAAAGAGAAGA/UC*^*L*^*UUUC*^*L*^*UCUUC*^*L*^*U*	D10	9.64	10.51	0.87	1.2	50	54	0.1	96	32	-2.00	-0.75
*AGAAAGAGAAGA*/U^M^C^M^U^M^U^M^U^M^C^M^U^M^C^M^U^M^U^M^C^M^U^M^	D11	8.33	8.86	0.53	0.7	71	46	1.2	68	47	-1.45	-0.70
*AGAAAGAGAAGA*/U^M^C^L^U^M^U^M^U^M^C^L^U^M^C^M^U^M^U^M^C^L^U^M^	D12	11.46	11.70	0.24	0.6	77	44	0.4	89	38	-0.95	1.40
A^M^G^M^A^M^A^M^A^M^G^M^A^M^G^M^A^M^A^M^G^M^A^M^	S3*	-	-	-	2.3^τD^	0	*R*_*0*_	3.6	0	*R*_*0*_	-	-
A^M^G^M^A^M^A^M^A^M^G^M^A^M^G^M^A^M^A^M^G^M^A^M^/TCTTTCTCTTCT	D13	6.50	6.81	0.31	1.3	43	56	1.4	62	50	-0.30	0.60
A^M^G^M^A^M^A^M^A^M^G^M^A^M^G^M^A^M^A^M^G^M^A^M^/TC^L^TTTC^L^TCTTC^L^T	D14	9.92	9.52	-0.40	1.0	54	52	0.9	74	45	-0.75	-0.40
A^M^G^M^A^M^A^M^A^M^G^M^A^M^G^M^A^M^A^M^G^M^A^M^/*UCUUUCUCUUCU*	D15	7.60	7.91	0.31	0.9	58	51	0.7	81	42	-1.65	-0.75
A^M^G^M^A^M^A^M^A^M^G^M^A^M^G^M^A^M^A^M^G^M^A^M^/*UC*^*L*^*UUUC*^*L*^*UCUUC*^*L*^*U*	D16	10.06	10.82	0.76	0.5	76	46	0.6	83	42	-0.80	0.65
A^M^G^M^A^M^A^M^A^M^G^M^A^M^G^M^A^M^A^M^G^M^A^M^/U^M^C^M^U^M^U^M^U^M^C^M^U^M^C^M^U^M^U^M^C^M^U^M^	D17	7.24	9.15	1.91	0.6	75	45	0.3	92	36	-0.30	0.00
A^M^G^M^A^M^A^M^A^M^G^M^A^M^G^M^A^M^A^M^G^M^A^M^/U^M^C^L^U^M^U^M^U^M^C^L^U^M^C^M^U^M^U^M^C^L^U^M^	D18	11.11	12.54	1.43	0.6	75	45	0.1	96	32	-0.45	1.25

^a^—solution: 40 mM boric acid, 40 mM phosphoric acid, 40 mM acetic acid, and 100 mM sodium chloride, pH 5.0 and pH 7.0;

^b^—the value of χ^2^≤ 1.2;

τ_DA_—fluorescence lifetimes of donor with acceptor; τ_D_—fluorescence lifetime of donor (references values); R_0_—Förster distance; R—distance between donor and acceptor fluorophores; h_1/2_—value of half high maximum peak at 220 nm and minimum peak at 210 nm;

*—S1, S2, S3 control probes for D1-D6, D7-D12 and D13-D18 duplexes, respectively. Meaning of used fonts: italic—RNA, A^M^, G^M^, U^M^ and C^M^—2’-O-Me RNA, C^L^—LNA cytidine.

### Spectroscopic features of duplexes

**Fig 2:**
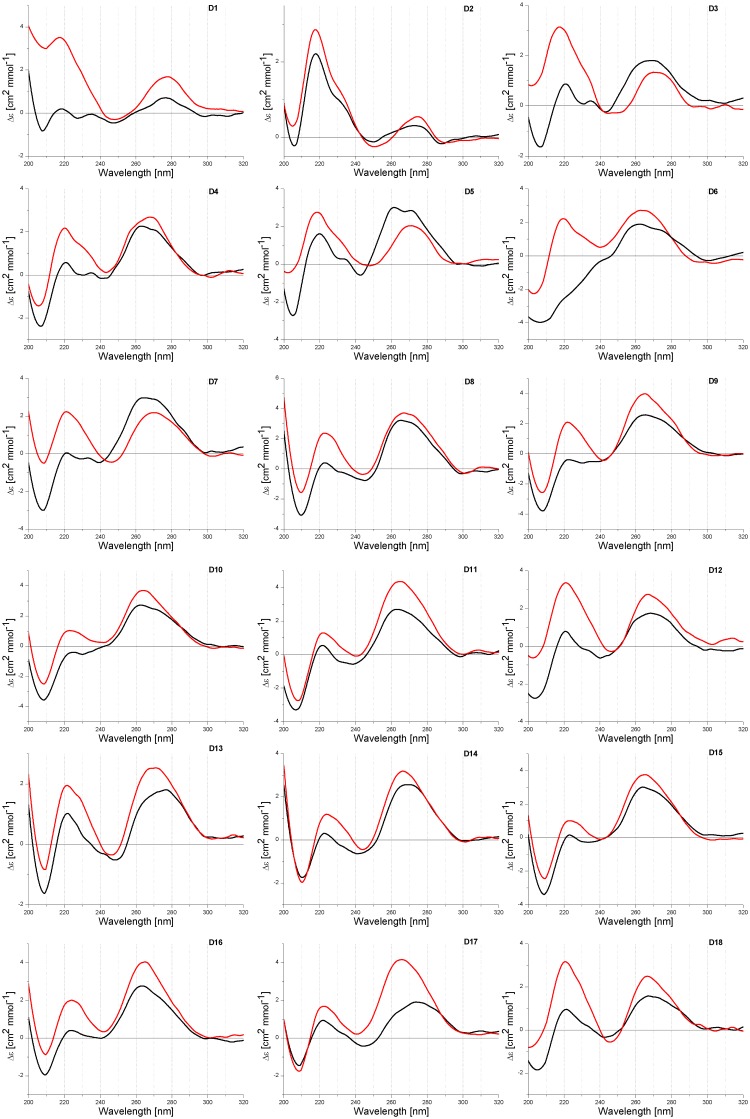
CD spectra of D1-D18 duplexes at pH 5.0 (black line) and 7.0 (red line).

The use of CD spectroscopy to study the geometry of DNA and RNA molecules is well established [21, 35]. CD spectra were performed at pH 5.0 and 7.0 in RB buffer containing 100 mM NaCl at 4°C (Table 1, Fig 2). In general, a positive long wavelength band at about 260–280 nm suggests the formation of right-handed helices. It is also a postulate that for DNA duplexes transition from positive to negative peak in the 210–220 nm range is a consequence of changing strand orientation from antiparallel to parallel [29]. For selected homopurine and homopyrimidine oligonucleotides the inversion of both strands was observed due to changing buffer pH value from 7.0 to 5.0. Moreover, LNA introduction into homopyrimidine oligonucleotide can facilitate reorientation of interacting strands and parallel duplex can form at neutral pH more efficiently due to their higher stability.

Among 18 recorded CD spectra of homopurine oligonucleotides in DNA, RNA and 2’-O-MeRNA series, the formation of D7-D18 duplexes containing RNA or 2’-O-Me RNA strands results in negative ellipticity in the 210–220 nm range (Fig 2). It significantly complicates determination reorientation positive peak to negative when changing buffer pH from neutral to acidic. For duplex DNA/DNA (D1) peak at ca. 210 nm inverts from positive to negative by decreasing the pH from 7.0 to 5.0. For remaining duplexes (D2-D18) those peaks inversion is not such clear and there are two peaks in the region of 200–220 nm, *i*.*e*. one positive peak with maximum at ca. 220 nm and second one at ca. 208 nm which is negative, except duplex D3. Positive and negative characters of those peaks is strongly dependent on buffer pH value. At pH 5.0 ellipticity at 220 nm is lower than at pH 7.0, whereas for the same buffers ellipticity at 210 nm becomes more negative.

Very informative is comparison of average ellipticity values (h_1/2_) between maximum peak at 220 nm and minimum peak at 210 nm (Table 1). Analysis of CD spectra of D1-D18 duplexes indicated that h_1/2_ values become more negative at pH 5.0 than at pH 7.0. As reported by Sugimoto, for DNA/DNA and DNA/DNA-LNA duplexes theirs parallel orientation correlates with negative ellipticity at 218 nm [29]. Different character of the helices formed by DNA and RNA strands (B helix, D1 vs A helix, D9) influences significantly on CD spectra. More negative character of peaks at 210 nm at pH 5.0 than at pH 7.0 suggests also a parallel orientation of duplexes formed by RNA and 2’-O-Me RNA and theirs LNA chimeric oligonucleotides.

The majority of CD spectra of RNA duplexes indicates formation of A-type helix. Moreover, heteroduplexes DNA/RNA (D3-D8, D13 and D14) form helices which deviate from a canonical A-RNA and B-DNA structures. It is probably due to the formation of helix A and B intermediate structures. The reduction of ellipticity peaks ca. 260 nm in buffer pH 5.0 (except D3) in comparison to physiological pH was observed. Moreover, CD studies revealed some different patterns of CD curves depending on presence of LNA modification.

### FRET efficiency and fluorescence lifetimes of model duplexes

**Fig 3:**
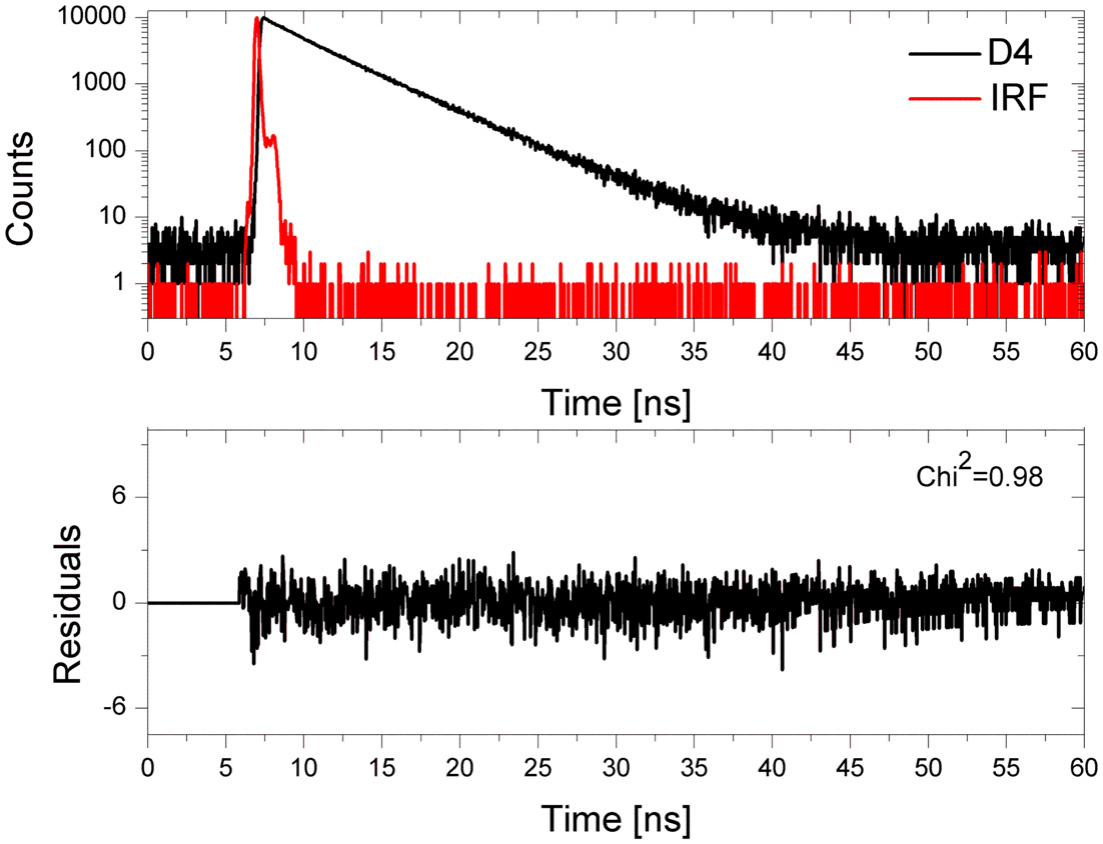
Time-resolved fluorescence lifetime analysis of the DNA/RNA-LNA (D4) duplex at pH 7.0. Top panel—donor fluorescence decay in the presence of acceptor (black line) and the instrument response function (IRF, red line); bottom panel—the weighted residuals.

**Fig 4:**
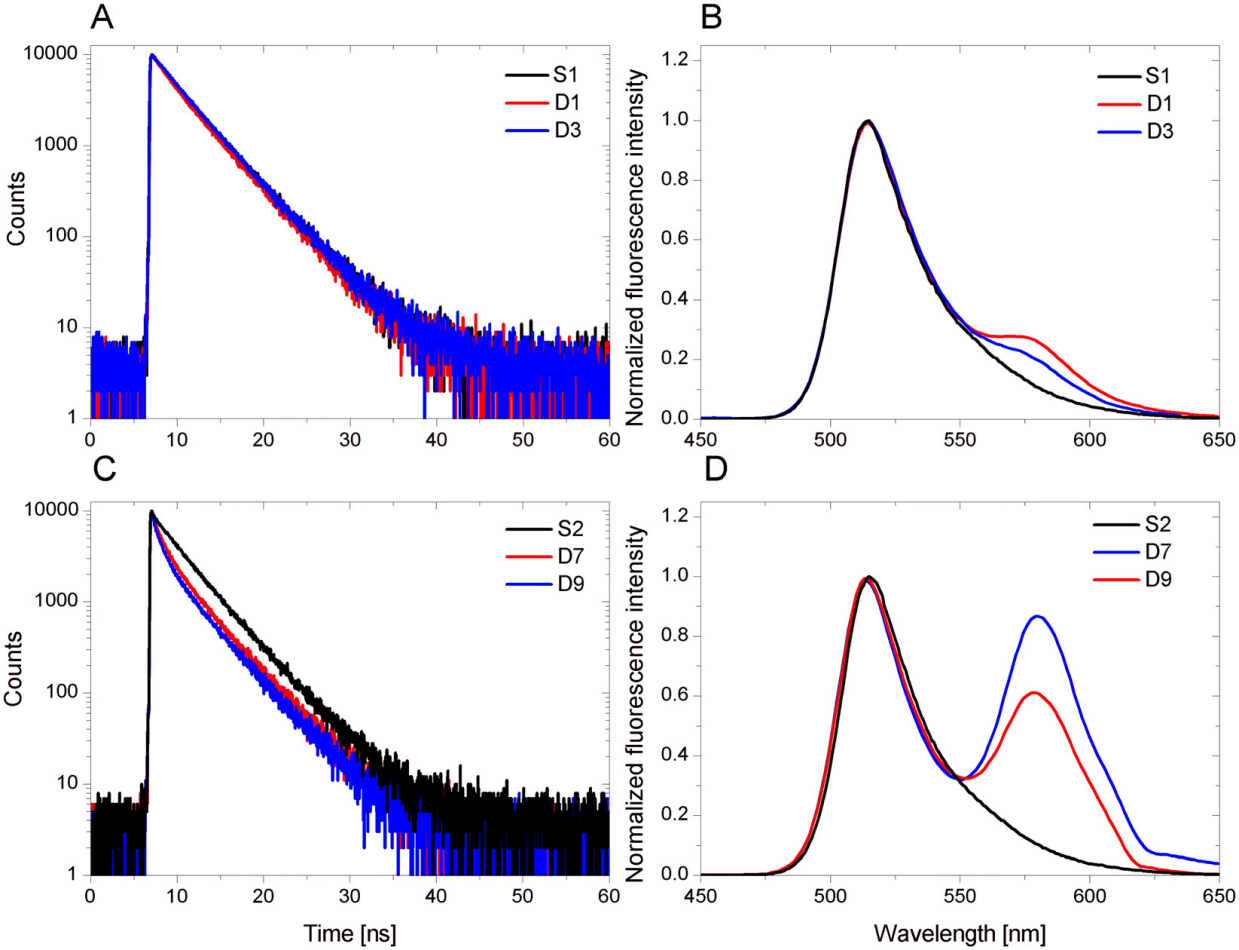
The fluorescence lifetime and normalized fluorescence intensity of selected D1, D3, D7 and D9 duplexes at pH 7.0. Black line indicates the S1 control probe for D1-D6 duplexes and the S2 control probe for D7-D12 duplexes. Red and blue lines indicate respective duplexes.

**Fig 5:**
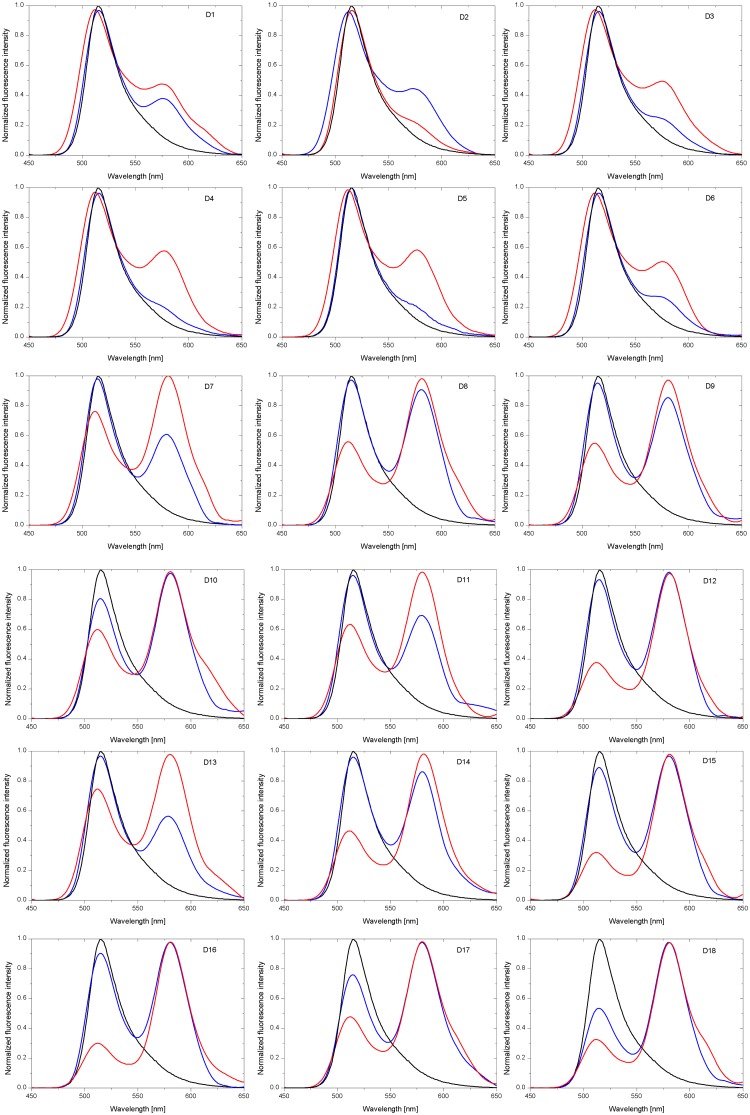
Normalized fluorescence intensity of D1-D18 duplexes at pH 7.0 (blue line) and pH 5.0 (red line). Black line indicates fluorescence of control probe S1 for D1-D6 duplexes, control probe S2 for D7-D12 duplexes as well as control probe S3 for D13-D18 duplexes.

In order to confirm that the model duplexes adopt a parallel orientation the efficiency of fluorescence resonance energy transfer (FRET) between 5,6-carboxyfluorescein (FAM) and 5-carboxytetramethylrhodamine (TAMRA) terminally attached to the 5’-end of the homopurine and homopyrimidine oligonucleotides, respectively, was studied. Common fluorophores pair, FAM as the donor (excitation at 494 nm and emission at 520 nm) and TAMRA as the acceptor (excitation at 565 nm and emission at 580 nm), also called the quencher was used [36]. In this system, changes of distance between FAM and TAMRA are due to the alteration of orientation from parallel to antiparallel by increasing the pH from 5.0 to 7.0 and they will correlate with efficiency of the energy transfer. The fluorescence intensity of FAM should decrease when the parallel duplex is formed and increase when the antiparallel structure is formed. That sequence/structure dependent energy transfer was observed for duplexes D1-D18 (Figs 3–5).

The fluorophores emission intensity depends primarily on their concentration, while the lifetime of the dye is mostly independent on the concentration. For this reason not only the steady-state fluorescence measurements but also the time-resolved fluorescence lifetime measurements were carried out. The first method was useful to qualitative analysis of FRET and the second one provided the data for determination of the energy transfer efficiency (*E*) (Fig 5 and Table 1).

Förster distance (*R*_*0*_) is the distance at which energy transfer efficiency is 50%. The *R*_*0*_ for FAM-TAMRA transfer was found to be 54±1 Å [37]. The values of energy transfer efficiency (E) were calculated from the measured fluorescence lifetime (Table 1) and are given by the following equation:

$$E=1-\frac{\tau_{DA}}{\tau_{D}}=\frac{1}{1+{\left(\frac{R}{R_{0}}\right)}^{6}}$$ (1)

where: τ_*D*_ is the fluorescence lifetime of donor (*i*.*e*. the measured lifetime of donor in the absence of acceptor), τ_*DA*_ is the fluorescence lifetime of donor with acceptor (*i*.*e*. the measured lifetime of donor in the presence of acceptor).

The distance between donor and acceptor fluorophores (*R*) in model duplexes was calculated from the energy transfer efficiency (*E*) and is given by the following equation:

$$R=R_{0}\sqrt[{6}]{\frac{1-E}{E}}$$ (2)

where: *R*_*0*_ is the Förster distance, *E* is the FRET efficiency [36].

Because *E* depends strongly on distance, measurements of the distance (*R*) are only reliable when *R* is within a factor of 2 of *R*_0_. If *R* is twice the Förster distance (*R* = 2*R*_*0*_) then the transfer efficiency is 1.54%, and if *R* = 0.5*R*_*0*_ then the efficiency is 98.5%. In other words, it is rather difficult to precisely calculate the distance when *R* is outside the 0.5*R* < *R*_0_ < 2*R* region due to much weaker dependence of *R* on the extremely low or high transfer efficiencies.

In general, all duplexes can be divided in two groups depending on the FRET efficiency (*E*) (Table 1). The first one with the values of *E* within the limits of confidence (understood as 0.5R_0_ and 2R_0_, *i*.*e*. 27Å<*R*<108Å) for D1, D5 and D7-D18 duplexes at pH 5.0 and D1-D4 as well as D8-D18 duplexes at pH 7.0, and the second one containing duplexes D2-D4 and D6 at pH 5.0 and D5-D6 at pH 7.0 that the values of *E is* outside the limits of confidence (understood as 27Å˃*R*˃108Å). The fluorescence lifetimes of duplexes with a homopurine DNA strand are almost the same as a control probe (S1) under all pH conditions. However, duplexes containing homopurine RNA strand show shorter lifetimes resulting in a high FRET efficiency at both pH values. These results confirm a parallel orientation of the strands in duplexes D7-D18. Interestingly, the presence of three LNA nucleotides within DNA as well as RNA strand causes decrease of fluorescence lifetimes at acidic pH for RNA/DNA-LNA (1.4 ns), RNA/RNA-LNA (1.2 ns), RNA/2’-O-Me RNA-LNA (0.6 ns) as well as 2’-O-Me RNA/DNA-LNA (1.0 ns) and 2’-O-Me RNA/RNA-LNA (0.5 ns) in comparison to duplexes without LNA moieties *i*.*e*. D7 (1.8 ns), D9 (1.6 ns), D11 (0.7 ns), D13 (1.3 ns) and D15 (0.9 ns), respectively. Similar behavior is also observed for LNA-modified duplexes at pH 7.0 what might be due to a higher thermal stability of these structures.

Fig 3 shows the fluorescence decay curve for DNA/RNA-LNA duplex along with the instrument response function (IRF), while Fig 4 illustrates the fluorescence lifetime as well as the normalized fluorescence intensity of selected duplexes (D1, D3, D7 and D9) at pH 7.0. The experimental data reveals some differences in the FRET efficiency between D1, D3, D7 and D9 duplexes. The fluorescence decay curves and the fluorescence emission spectra indicate that the energy transfer is noticeably more efficient for RNA/DNA (D7) and RNA/RNA (D9) than for DNA/DNA (D1) and DNA/RNA (D3) duplexes. One can see a distinct increase in the fluorescence intensity of TAMRA at around 580 nm for D7 and D9 duplexes (Fig 4D), while a small peak appears near 580 nm in case of D1 and D3 duplexes (Fig 4B). Moreover, D1 and D3 fluorescence decays are virtually the same as for a control sample (S1) in the absence of a donor (Fig 4A). In contrast, the lifetimes of D7 and D9 are shorter in regard to the control probe (S2) (Fig 4C). Significant shortening of a fluorescence lifetime is particularly noticeable in an initial phase of fluorescence decay due to the logarithmic scale of the decay trace.

Due to the fact that FRET parameters calculated based on time-resolved fluorescence measurements (Table 1) are consistent with the fluorescence spectra obtained in steady-state fluorescence assays (Fig 5), it suggests that duplexes can adopt parallel strand orientation under experimental conditions.

## Discussion

### Thermodynamic features of duplexes

Based on the thermodynamic parameters, it was observed that stability of DNA homo- and heteroduplexes are lower than respective 2’-O-Me RNA and RNA duplexes. Moreover, the results indicate that presence of LNA within DNA, RNA and 2’-O-Me RNA duplexes enhances their thermal stability and the strongest stabilities enhancement was observed for latter ones (Table 1 and S1 Table). These results stay in accordance with the data of Kierzek group [28, 38].

Analysis of thermodynamic stabilities of the same duplexes in buffers pH 5.0 and 7.0 indicated that DNA homo- and heteroduplexes are more stable at pH 5.0, however, the melting process is non two-state (S1 Table, D1-D6). Enhancement stabilities (ΔΔG°_37_) oscillates between 1.6 and 5.4 kcal/mol. When purine strands were RNA (D7-D12) or 2’-O-Me RNA (D13-D18), both homo- and heteroduplexes performed very similar thermodynamic stabilities (except D17 and D18) in both buffers. In most cases, RNA and 2’-O-Me RNA homo- and heteroduplexes (D13-D18) are less stable at pH 5.0 than at pH 7.0. This difference of stabilities ranges between 0.3 and 0.9 kcal/mol. The exceptions concern duplexes D17 and D18 where differences of the thermodynamic stabilities were 1.9 and 1.4 kcal/mol, respectively. For D14 only, it was observed more favorable stability (ΔΔG°_37_ equal to 0.4 kcal/mol) in acidic condition. Furthermore, presence of LNA moieties within one strand of DNA and RNA duplexes enhance their thermodynamic stability. Additionally, when LNA units were introduced in the 2’-O-Me RNA strand, the stabilizing effect was measurably stronger. These results are in accordance with data in the literature [35, 38, 39].

The influence of transition from antiparallel to parallel orientation in RNA and 2’-O-Me RNA duplexes on their thermodynamic stability was not reported. It is postulated that parallel duplex is formed by reverse Watson-Crick pairs and such base pairs was confirmed by NMR and Raman spectroscopies and chemical methylation [7]. Different stability of DNA, 2’-O-Me RNA and RNA homo- and heteroduplexes at pH 5.0 and 7.0 can be the result of theirs reorientation to parallel orientation and/or topology of B-DNA and A-RNA helices. To the best of our knowledge only paper published by Sugimoto concerns DNA duplexes and the influence of the presence of several LNA nucleotides in homopyrimidine strand [29]. Also Hrdlicka reported that incorporation of 2’-amino-β-L-LNA into α-DNA strands results in formation of stable duplexes with complementary RNA in parallel orientation [40]. Moreover, Miyoshi et al. demonstrated that the formation of the parallel DNA duplexes were significantly and specifically stabilized by polylysine comb-type copolymer with hydrophilic graft chain. This polymer led to greater stabilization of the parallel-stranded DNA duplex than the antiparallel duplex under certain conditions [41].

Reported herein data showed that parallel DNA duplexes are more stable than antiparallel whereas stability of RNA and 2’-O-Me RNA based duplexes was very similar in both buffers. Topology of DNA and RNA duplexes is different and possibly observed thermodynamic properties correspond to those differences.

### Circular dichroism spectroscopy features of duplexes

Comparison of CD spectra of DNA (D1) and RNA (D9) homoduplexes at pH 5.0 and 7.0 reveals differences which are related with B and A helix geometry, respectively (Fig 2). It indicates high sensitivity of CD spectroscopy to duplex structure changes. Character of CD spectra of the D1-18 duplexes in the 240 and 300 nm range showed their similarities. A long-wavelength positive peak with maximum near 265 nm was observed, however, the intensity of this peak is different for particular duplexes and it is shifted by a few nm. A larger differentiation of CD spectra is observed in the 200 and 240 nm range. For most duplexes, positive peak ca. 220 nm and negative peak ca. 210 nm were observed. Major difference concerns the intensity of both peaks and in consequence, a various character of the spectra in that range is observed. Very characteristic is peak ca. 210 nm which is mostly negative, exceptions are DNA duplexes (D1- D3). Comparison of the values of half high (h_1/2_) maximum peak at 220 nm and minimum peak at 210 nm indicates that these values are lower (more negative) at pH 5.0 than at pH 7.0 for all analyzed duplexes (Table 1).

Sugimoto published CD spectra of duplexes D1 and D2, and postulate that negative value of peak at 210 nm (Sugimoto reported this peak as 218 nm) is due to the parallel orientation of DNA duplexes [29]. CD spectra of 2’-O-Me RNA and RNA homo- and heteroduplexes were not reported. By analogy to change of reported CD spectra and correlation of those changes with parallel orientation of DNA/DNA and DNA/DNA-LNA duplexes, it is reasonable to assume that more negative character peak at 210 nm at pH 5.0 is also correlated with parallel orientation of duplexes. Fluorescence studies presented herein demonstrate that in most cases antiparallel and parallel duplexes exist simultaneously, however, ratio of both type of duplexes is different and it depends on duplex composition and buffer pH. As consequence of that, recorded CD spectra presumably reflect simultaneous the presence of differently oriented duplexes.

### Fluorescence spectroscopy features of duplexes

Various FRET-based techniques are commonly used to determine molecular distances and interactions within labeled nucleic acid systems [42]. Fluorescence quenching experiments can provide reliable proof of formation of duplexes. Therefore, conformational changes of DNA and RNA duplexes were studied in this work using two fluorescence assays, *i*.*e*. steady-state and time-resolved fluorescence methods. Obtained results revealed that fluorescence intensities and lifetimes of duplexes containing homopurine DNA strand do not change at pH 5.0 and 7.0.

FRET parameters for all DNA complexes are virtually identical and this indicates mostly antiparallel orientation of the strands (Table 1, D1-D6). However, structures formed with RNA homopurine strands exhibit different fluorescence characteristics (Fig 5, D7-D18). On the basis of these results structural transition of RNA complexes from antiparallel to parallel conformation is suggested. Furthermore, it was observed that the presence of three LNA-C within one strand of duplex causes increase the acceptor fluorescence intensity and shortening of the fluorescence lifetime, what can be explained by a higher thermal stability of modified structures.

It must be emphasized that although theoretical values of FRET efficiencies were calculated based on lifetime measurements, in practice, many fluorescence decays are more complex. Excited molecules are often in an inhomogeneous environment and quenching processes can lead to multiexponential decay behavior. Moreover, the influence of any event on the distance between donor and acceptor molecules will affect the resonance energy transfer rate. Therefore, the numbers (*i*.*e*. calculated distances) shown in Table 1 should not be treated as absolute values. On the other hand, one can easily notice the difference between highly-efficient FRET structures and the structures where FRET efficiencies were negligibly small (Fig 5).

## Conclusions

Combination of synthetic chemistry, thermodynamic UV-melting analysis, circular dichroism and fluorescence spectroscopies has allowed to obtain a global view of formation parallel and antiparallel duplexes by unmodified and modified DNA, 2’-O-Me RNA and RNA oligonucleotides. The present results demonstrate that homo- and heteroduplexes could be characterized by those biophysical methods to search theirs antiparallel and parallel orientation. However, very limited number of this type of the investigations published in literature makes unambiguous conclusions difficult. Herein the results concern CD spectra, broad thermodynamic studies and also fluorescence analyzes of 18 various duplexes.

The fluorescence assays in which AGAAAGAGAAGA and its 2’-O-methylated analog was used, indicated that for some duplexes a significant number of parallel duplexes is present even at pH 7.0. Moreover, the changing of conditions from neutral to acidic results in shifting equilibrium towards parallel form of duplex. This dynamic equilibrium is very dependent on type of strand which forms duplex and buffer pH values.

It is difficult to interpret ambiguously thermodynamic results. The most surprising is large difference in stabilities of DNA and RNA type duplexes at pH 5.0 and 7.0. DNA duplexes at pH 5.0 are 2–4 kcal/mol more stable whereas 2’-O-Me RNA and RNA duplexes are up to 2.0 kcal/mol less stable. Presumably, it correlates with different geometry of DNA and RNA duplexes and in consequence different hydrogen bonds and base stacking interactions including inter- and intrastranded interactions. Detailed thermodynamic and structural characteristics of model duplexes can provide useful information to design potential antisense tools.

## Supporting Information

S1 Fig UV melting curves of model DNA, RNA and 2’-O-MeRNA duplexes at pH 5.0. (TIF)

S2 Fig UV melting curves of model DNA, RNA and 2’-O-MeRNA duplexes at pH 7.0. (TIF)

S1 Table Thermodynamic parameters of model duplexes. a—solution: 40 mM boric acid, 40 mM phosphoric acid, 40 mM acetic acid, and 100 mM sodium chloride, pH 5.0 and pH 7.0; b—calculated for 10^−4^ M oligomer concentration. Values in parenthesis concerns stabilities calculated form fitting melting curves whereas the values above were derived from correlation of melting temperature and duplex concentration.
(DOC)
