# Supplementary material for: Structural Aspects of the Antiparallel and Parallel Duplexes Formed by DNA, 2’-O-Methyl RNA and RNA Oligonucleotides
Source: PLoS One. 2015 Nov 18;10(11):e0143354. doi: 10.1371/journal.pone.0143354 (PMC4666348; doi:10.1371/journal.pone.0143354)
Supplement: S1 Table — a—solution: 40 mM boric acid, 40 mM phosphoric acid, 40 mM acetic acid, and 100 mM sodium chloride, pH 5.0 and pH 7.0; b—calculated for 10−4 M oligomer concentration. Values in parenthesis concerns stabilities calculated form fitting melting curves whereas the values above were derived from correlation of melting temperature and duplex concentration. (DOC) [file pone.0143354.s003.doc]

**S1 Table** **Thermodynamic parameters of model duplexes.** a – solution: 40 mM boric acid, 40 mM phosphoric acid, 40 mM acetic acid, and 100 mM sodium chloride, pH 5.0 and pH 7.0; b – calculated for 10^-4^ M oligomer concentration. Values in parenthesis concerns stabilities calculated form fitting melting curves whereas the values above were derived from correlation of melting temperature and duplex concentration.

**S1 Table Thermodynamic parameters of model duplexes.^a^**

| **Name of duplexes** | **pH 5.0, thermodynamic parameters from T_M_^-1^ vs log C_T_ plots**  **(average of curve fits)** | | | | | **pH 7.0, thermodynamic parameters from T_M_^-1^ vs log C_T_ plots**  **(average of curve fits)** | | | | |  |
| --- | --- | --- | --- | --- | --- | --- | --- | --- | --- | --- | --- |
|  | -ΔH°  (kcal/mol) | -ΔS°  (eu) | -ΔG°_37_ (kcal/mol) | T_M_^b^  (°C) | ΔΔG°_37_ (kcal/mol) | -ΔH°  (kcal/mol) | -ΔS°  (eu) | -ΔG°_37_ (kcal/mol) | T_M_^b^  (°C) | ΔΔG°_37_ (kcal/mol) | ΔΔG°_37_ (pH 5.0) - ΔΔG°_37_(pH 7.0)  (kcal/mol) |
| D1 | 48.2±1.0  (99.5±12.7) | 135.3±3.2  (304.7±40.3) | 6.25±0.03  (5.02±0.05) | 35.2  (32.4) | 0 | 51.4±3.9  (55.4±6.9) | 150.6±13.2  (164.2±23.2) | 4.67±0.17  (4.45±0.32) | 26.1  (25.8) | 0 | -1.58±0.17 |
| D2 | 43.0±0.7  (90.2±7.7) | 112.5±2.3  (263.2±23.7) | 8.07±0.01  (8.61±0.48) | 48.5  (44.3) | -1.82 | 59.5±13.8  (57.1±7.9) | 178.7±46.4  (170.4±26.5.) | 4.11±0.85  (4.22±0.42) | 24.9  (24.9) | 0.56 | -3.96±0.85 |
| D3 | 51.5±1.1  (120.4±12.5) | 144.1±3.5  (368.4±38.4) | 6.77±0.01  (6.10±0.73) | 38.5  (35.9) | 0 | 46.3±4.1  (77.0±5.7) | 138.9±14.5  (246.3±18.8) | 3.27±0.38  (0.59±0.23) | 16.6  (14.8) | 0 | -3.50±0.38 |
| D4 | 62.9±2.2  (119.9±4.7) | 172.4±6.8  (350.2±14.3) | 9.42±0.07  (11.30±0.58) | 51.9  (49.8) | -2.65 | 44.6±1.9  (79.7±6.8) | 126.9±6.5  (245.7±21.4) | 5.26±0.11  (3.49±0.19) | 28.4  (25.6) | -1.99 | -4.16±0.13 |
| D5 | 50.9±1.0  (103.6±10.4) | 139.5±3.4  (308.7±31.1) | 7.70±0.01  (7.87±0.84) | 44.1  (41.1) | 0 | 42.4±9.3  (44.8±20.2) | 125.8±32.6  (133.5±71.1) | 3.42±0.99  (3.43±1.88) | 15.8  (16.9) | 0 | -4.28±0.99 |
| D6 | 62.7±1.7  (93.6±8.1) | 168.9±5.3  (264.0±24.6) | 10.35±0.08  (11.78±0.61) | 57.1  (55.4) | -2.65 | 43.0±2.7  (67.9±6.1) | 120.0±9.2  (193.0±20.0) | 5.81±0.12  (5.00±0.26) | 31.9  (29.8) | -2.39 | -4.54±0.14 |
| D7 | 64.4±2.6  (68.6±5.5) | 186.9±8.7  (200.5±17.8) | 6.44±0.06  (6.39±0.08) | 36.6  (36.4) | 0 | 71.9±1.2  (71.4±1.9) | 208.9±3.8  (207.3±6.1) | 7.12±0.01  (7.14±0.04) | 39.6  (39.7) | 0 | 0.68±0.06 |
| D8 | 75.5±3.6  (74.1±2.0) | 213.7±11.6  (209.5±6.6) | 9.18±0.08  (9.16±0.11) | 48.3  (48.4) | -2.74 | 82.8±4.6  (85.1±1.5) | 234.9±14.2  (242.2±5.0) | 9.94±0.06  (10.02±0.16) | 50.3  (50.3) | -2.82 | 0.76±0.10 |
| D9 | 61.6±2.0  (81.9±9.5) | 174.2±6.6  (240.1±30.4) | 7.58±0.03  (7.47±0.17) | 42.4  (40.6) | 0 | 74.1±1.2  (73.6±2.8) | 213.0±4.0  (211.4±8.8) | 7.99±0.01  (8.00±0.05) | 43.2  (43.3) | 0 | 0.41±0.03 |
| D10 | 76.3±4.0  (73.2±6.9) | 215.0±12.7  (205.4±21.3) | 9.64±0.13  (9.56±0.33) | 50.2  (50.4) | -2.06 | 80.0±1.4  (84.1±2.9) | 224.0±4.4  (236.7±9.1) | 10.51±0.06  (10.67±0.11) | 53.2  (53.1) | -2.52 | 0.87±0.14 |
| D11 | 63.5±1.1  (68.0±3.8) | 176.7±3.6  (192.1±12.0) | 8.33±0.01  (8.40±0.10) | 46.1  (45.8) | 0 | 68.9±0.7  (70.6±5.1) | 193.7±2.4  (198.8±16.0) | 8.86±0.01  (8.93±0.10) | 47.8  (47.9) | 0 | 0.53±0.01 |
| D12 | 81.2±10.2  (85.6±6.9) | 224.9±31.4  (238.4±21.5) | 11.46±0.58  (11.69±0.46) | 57.0  (56.9) | -3.13 | 79.3±7.2  (83.0±12.7) | 218.0±22.0  (229.0±38.4) | 11.70±0.39  (11.96±0.84) | 58.6  (58.7) | -2.84 | 0.24±0.70 |
| D13 | 60.1±1.6  (67.5±3.4) | 172.8±5.3  (197.2±11.1) | 6.50±0.03  (6.37±0.09) | 36.9  (36.2) | 0 | 68.2±2.4  (68.1±2.7) | 197.8±7.9  (197.6±8.8) | 6.81±0.04  (6.80±0.06) | 38.3  (38.2) | 0 | 0.31±0.03 |
| D14 | 86.6±8.5  (73.4±2.8) | 247.2±26.7  (205.9±8.9) | 9.92±0.28  (9.55±0.20) | 49.6  (50.3) | -3.42 | 71.0±1.8  (73.5±3.8) | 198.1±5.6  (206.0±11.9) | 9.52±0.05  (9.59±0.13) | 50.7  (50.5) | -2.71 | -0.40±0.28 |
| D15 | 66.2±2.3  (70.8±2.8) | 188.9±7.4  (203.7±8.7) | 7.60±0.02  (7.63±0.07) | 42.1  (41.9) | 0 | 69.7±2.4  (70.1±4.0) | 196.0±7.8  (200.4±13.0) | 7.91±0.02  (7.96±0.11) | 43.4  (43.4) | 0 | 0.31±0.02 |
| D16 | 65.2±2.8  (85.1±9.8) | 177.7±8.6  (239.4±29.6) | 10.06±0.11  (10.90±0.63) | 54.8  (53.8) | -2.46 | 80.1±0.3  (79.6±7.8) | 223.3±1.0  (221.7±24.0) | 10.82±0.01  (10.86±0.39) | 54.6  (54.9) | -2.91 | 0.76±0.11 |
| D17 | 80.0±7.0  (72.6±2.7) | 234.7±22.8  (210.5±8.9) | 7.24±0.18  (7.36±0.13) | 39.8  (40.6) | 0 | 63.7±2.4  (67.8±3.2) | 175.9±7.5  (188.9±10.0) | 9.15±0.05  (9.24±0.14) | 50.3  (49.9) | 0 | 1.91±0.19 |
| D18 | 68.4±10.0  (72.6±2.0) | 184.6±30.4  (197.5±5.9) | 11.11±0.64  (11.37±0.18) | 59.3  (59.1) | -3.87 | 85.7±22.0  (78.2±1.9) | 234.0±67.4  (212.8±5.8) | 12.54±1.22  (12.20±0.08) | 60.4  (61.2) | -3.39 | 1.43±1.38 |

a – solution: 40 mM boric acid, 40 mM phosphoric acid, 40 mM acetic acid, and 100 mM sodium chloride, pH 5.0 and pH 7.0; b – calculated for 10^-4^ M oligomer concentration. Values in parenthesis concerns

stabilities calculated form fitting melting curves whereas the values above were derived from correlation of melting temperature and duplex concentration.
